# Supplementary material for: ‘Stray appetites’: a socio-ecological analysis of free-ranging dogs living alongside human communities in Bangalore, India
Source: Urban Ecosyst. 2021 Feb 16;24(6):1245–58. doi: 10.1007/s11252-021-01097-4 (PMC8551108; doi:10.1007/s11252-021-01097-4)
Supplement: Supplementary file 1 — (PDF 863 kb) [file 11252_2021_1097_MOESM1_ESM.pdf]

ESM

'Stray Appetites': A Socio-Ecological Analysis of Free-Ranging Dogs Living Alongside Human Communities in Bangalore, India.

Shireen Jagriti Bhalla[1*], Roy Kemmers[1†], Ana Vasques[1†], Abi Tamim Vanak[2,3,4†]

*Corresponding author: sjagritib@gmail.com

† Equal contributers

[1]Erasmus University College, Rotterdam, the Netherlands

[2]Ashoka Trust for Research in Ecology and the Environment, Bengaluru, India

[3]DBT/Wellcome Trust India Alliance Fellow, Hyderabad, India

[4]School of Life Sciences, University of KwaZulu-Natal, Durban, South Africa

Survey area

a. b. c.

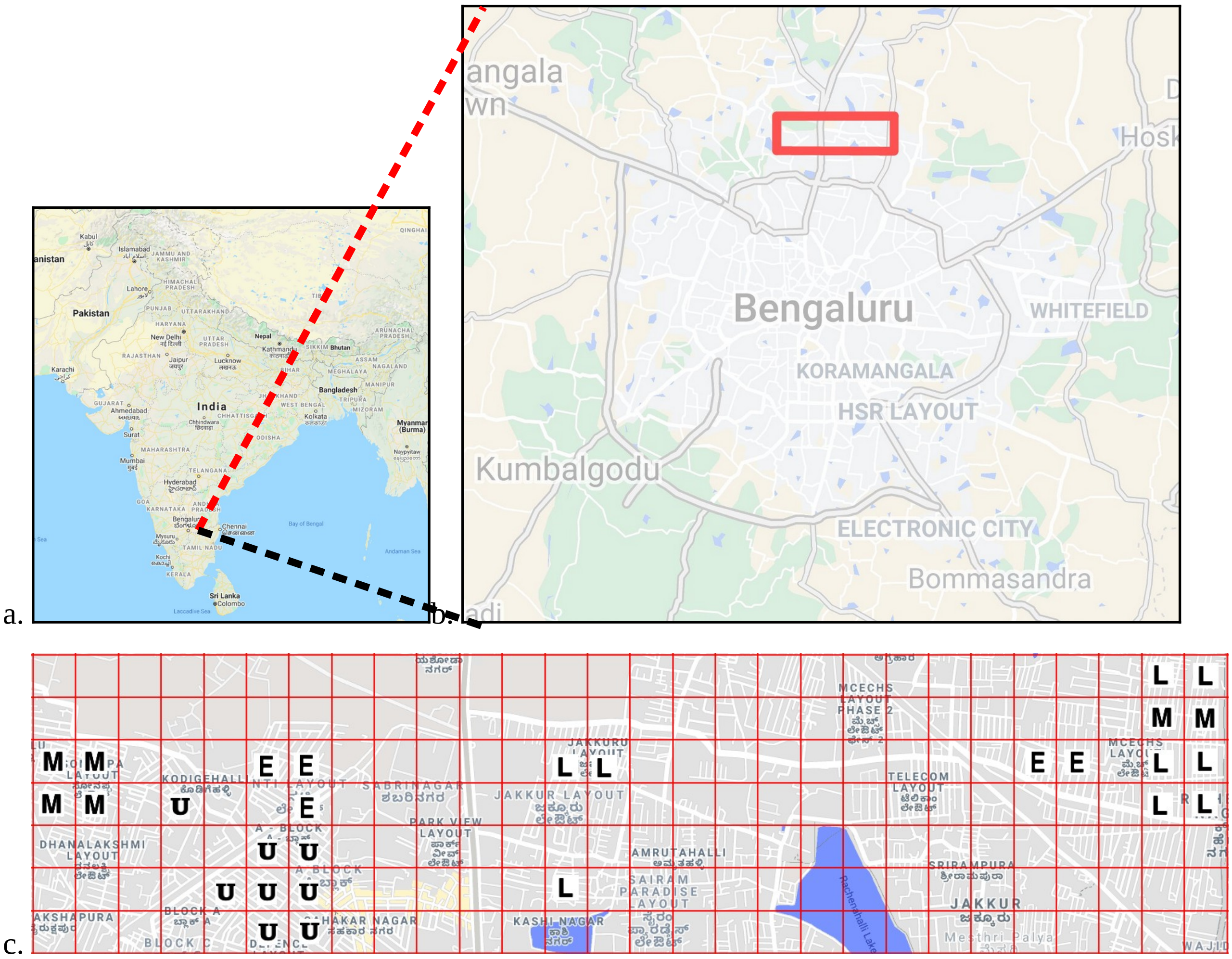

Fig. S1 a. Position of Bangalore city on India map (Accessed from GoogleMaps). b. Area in North Bangalore, India where both surveys of free-ranging dog populations and qualitative surveys of residents were conducted for this study. c. Units of area 248*248m^2 where free-ranging dog populations were surveyed by photographic capture-recapture Spread of sample units across North Bangalore is depicted with each unit characterised; L: lower SE class, M: middle SE class, U: upper SE class, and E: empty unit (relatively undeveloped area) .

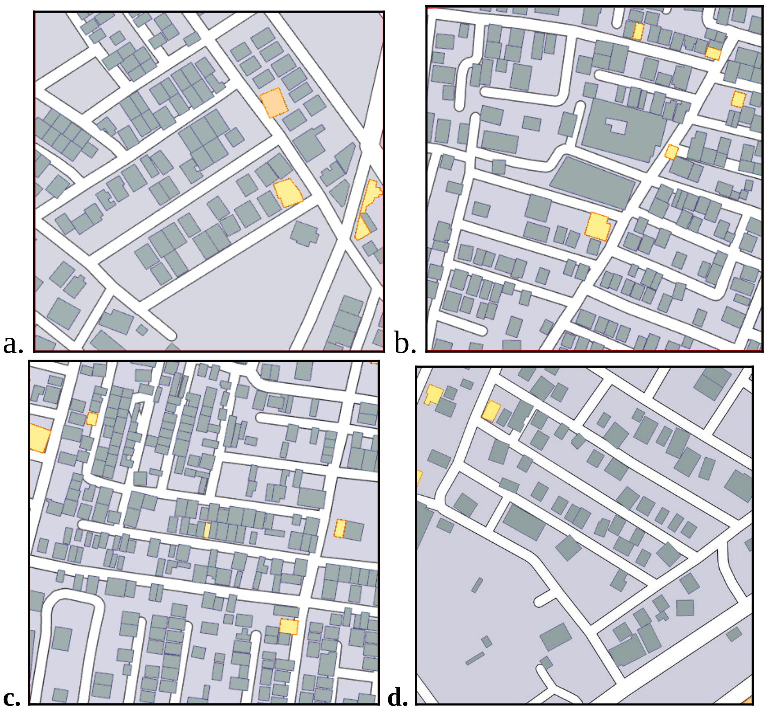

Fig S2. Illustration of typical housing density by unit charecterisation: a. Upper SE class, b. Middle SE class, c. Lower SE class, d. Empty unit.

Ecology Data Tables

Table S1. Free-ranging dog population numbers in sampled areas

Grid Reference	#Dogs sighted	#Uniques	#Samples	Population estimate (from Superduplicates)	se of population estimate
1	14	9	4	24.16	8.63
2	21	9	4	27.04	4.28
3	38	19	4	52.92	7.35
4	65	27	4	82.54	7.86
5	32	16	4	44.54	6.69
6	9	7	4	21.68	11.45
7	15	5	4	17.7	2.66
8	14	7	4	19.46	4.19
9	11	6	3	15	4.81
10	12	4	4	14.16	2.55
11	11	5	4	14.54	4.1
12	18	4	4	19.65	2.01
13	12	5	4	15.26	3.88
14	26	12	4	34.63	5.36
15	14	9	4	24.12	10.92
16	9	5	4	13.48	5.56
17	23	11	4	31.22	5.27
18	32	12	4	39.13	4.06
19	11	3	4	12.41	1.81
20	16	3	4	17.12	1.46
21	28	12	4	36.06	4.79
22	37	10	4	41.67	3.28

23	50	21	4	63.76	5.72
24	95	37	4	117.69	8.11
25	42	19	4	55.36	6.4
26	45	22	4	61.84	7.95
27	26	9	4	31.01	3.47
28	35	16	4	46.4	5.86

Table S2. Population and food source data of areas sampled

Grid #	Chao2.est	Garbage	Shops	Bakeries	Butchers	Restaurants	Houses	SE class
1	24.16	2	1	0	0	0	113	M
2	27.04	1	1	0	0	0	176	M
3	52.92	1	2	1	0	0	182	L
4	82.54	2	7	2	1	1	229	L
5	44.54	6	4	0	0	0	143	L
6	21.68	2	1	0	0	0	144	U
7	17.7	1	2	0	0	0	112	U
8	19.46	0	3	1	0	0	126	U
9	15	0	0	0	0	0	111	U
10	14.16	2	1	0	0	3	125	U
11	14.54	3	0	1	0	1	88	U
12	19.65	1	1	0	0	0	189	U
13	15.26	3	2	0	0	0	75	E
14	34.63	0	1	0	0	0	24	E
15	24.12	3	1	0	0	0	25	E

16	13.48	4	0	1	0	0	89	U
17	31.22	0	1	1	0	0	180	M
18	39.13	1	3	0	0	0	157	M
19	12.41	1	0	0	0	0	67	E
20	17.12	3	0	0	0	0	46	E
21	36.06	0	0	2	1	3	135	L
22	41.67	0	6	1	1	5	287	L
23	63.76	2	16	1	1	3	389	L
24	117.69	6	21	5	15	7	315	L
25	55.36	9	3	0	0	1	184	M
26	61.84	11	5	1	0	2	247	M
27	31.01	5	8	0	1	0	244	L
28	46.4	11	5	0	0	3	177	L

Descriptive Analysis of the Demographic Surveyed

Table S3. Sex descriptives of survey respondents

Gender	Frequency	Percent	Valid Percent	Cumulative Percent
Female	49	50.5	50.5	50.5
Male	48	49.5	49.5	100.0
Total	97	100.0	100.0	

Table S4. Age descriptives of survey respondents

Age category	Frequency	Percent	Valid Percent	Cumulative Percent
18-24	3	3.1	3.1	3.1
25-34	9	9.3	9.3	12.4
35-44	17	17.5	17.5	29.9
45-54	33	34.0	34.0	63.9
55-64	20	20.6	20.6	84.5
65-74	11	11.3	11.3	95.9
75-84	4	4.1	4.1	100.0
Total	97	100.0	100.0	

Table S5. Class descriptives of survey respondents

Socio-economic class	Frequency	Percent	Valid Percent	Cumulative Percent
Lower	20	20.6	20.6	20.6
Lower middle	9	9.3	9.3	29.9
Middle	16	16.5	16.5	46.4
Upper middle	12	12.4	12.4	58.8
Upper	40	41.2	41.2	100.0
Total	97	100.0	100.0	

Survey questions

Demographic Variables:

1. Sex
2. Age

Economic background:

3. What is the job of the main earner of your family?
4. What is your type of residence?
5. Is your house owned or rented?
6. Which locality do you live in?

Opinion on dogs:

7. To what extent do you agree with the following statement: There is a stray dog menace that needs to be solved by the city administration. *1-5 likert scale from 'Disagree strongly to Agree strongly'.*
8. To what extent do you agree with the following statement: Stray dogs are not a problem and should be left alone by the city administration. *1-5 likert scale from 'Disagree strongly to Agree strongly'.*
9. Please **elaborate** on your opinion about stray dogs
10. Do you think stray dogs should be removed from our cities? a. *Yes* b. *No* c. *Maybe* d. *Other.*
11. Please **elaborate**.
12. If yes, how do you think they should be removed? a. *ABC program* (gradual sterilisation over years) b. *Mass culling* c. ***Other***.

Contact with dogs:

13. Do you feed stray dogs? a. *Yes* b. *No* c. *No, but I support those who do.*
14. Have you now or previously had pet dogs at home? a. *Yes* b. *No.*
15. For the most part, how do you get to work/school? a. *Walk* b. *Cycle* c. *Motorised two-wheeler* d. *Car* e. *Bus* f. *Train* g. *Other.*
16. Have you or has someone close to you (family or friend) ever been: a. *Bitten* b. *Chased repeatedly*, by a stray dog?

Mean differences between classes

Table S6. ANOVA of mean differences between socioeconomic units on % households that feed stray dogs, dog population and number of houses

		Sum of Squares	df	Mean Square	F	Sig.
% of Households That Feed Stray Dogs	Between Groups	292.513	2	146.257	6.757	.006
	Within Groups	432.888	20	21.644		
	Total	725.402	22			
Dogs	Between Groups	6935.406	2	3467.703	9.516	.001
	Within Groups	7288.392	20	364.420		
	Total	14223.798	22			
Houses	Between Groups	51786.771	2	25893.385	6.955	.005
	Within Groups	74459.056	20	3722.953		
	Total	126245.826	22			
